# Breathing in vitro: Designs and applications of engineered lung models

**DOI:** 10.1177/20417314211008696

**Published:** 2021-04-28

**Authors:** Roberta Nossa, Joana Costa, Ludovica Cacopardo, Arti Ahluwalia

**Affiliations:** University of Pisa, Pisa, Italy

**Keywords:** Lung models, in vitro models, aerosol exposure, fluidic systems, stretching systems

## Abstract

The aim of this review is to provide a systematic design guideline to users, particularly engineers interested in developing and deploying lung models, and biologists seeking to identify a suitable platform for conducting in vitro experiments involving pulmonary cells or tissues. We first discuss the state of the art on lung in vitro models, describing the most simplistic and traditional ones. Then, we analyze in further detail the more complex dynamic engineered systems that either provide mechanical cues, or allow for more predictive exposure studies, or in some cases even both. This is followed by a dedicated section on microchips of the lung. Lastly, we present a critical discussion of the different characteristics of each type of system and the criteria which may help researchers select the most appropriate technology according to their specific requirements. Readers are encouraged to refer to the tables accompanying the different sections where comprehensive and quantitative information on the operating parameters and performance of the different systems reported in the literature is provided.

## Introduction

Epithelial barriers regulate the passage from one domain to another, and are the body’s natural defense against external substances.^1^ Lung epithelium is one of the most permeable epithelial barriers of the human body^2^ and it is the object of different investigations regarding drug and nanoparticle (NP) delivery and toxicology. Recent developments in delivering drugs to the lung are driving the need for studies to evaluate the fate of inhaled medicines.^3^ In particular, inhalation of aerosolized drugs is a promising route for noninvasive targeted drug delivery to the lung.^4^ Additionally, researchers are focusing their attention on the adverse effects caused by inhaled nanoparticles and chemical compounds (which depend on their hazard), and on exposure.^2^ To understand what can and cannot cross the lung barrier and their effects on the human tissues, models have emerged to rigorously study and investigate these questions.

Both in vivo and in vitro models are used for lung pathology (such as infection, inflammation, cancer, small-airway pulmonary diseases), drug delivery, and toxicology studies. Indeed, animal models provide a means for testing hypotheses, such as the therapeutic efficacy of a drug candidate, in complex biological systems. In vivo models are important for the evaluation of drug deposition efficiency, or to study the effects of nanomaterials and inhaled chemicals on lungs and peripheral tissue.^2,5–8^ However, although they can recapitulate key pathological changes in some lung diseases, they are still limited in reiterating all features observed in humans due to fundamental differences in anatomy and physiology between humans and animals. The combination of differences in host immune responses to epithelial injury, pathology biomarkers, the extent of respiratory bronchioles, interdigitation of conducting airways, acinar size, and air-blood barrier thickness contribute to the varied sensitivity to inhaled toxicants between species.^9^ In addition to differences in lung physiology and responses to compounds, animal testing is also a sensitive topic from an ethical point of view and a transition to non-animal technologies is encouraged through national legislation.

In vitro models offer tightly controlled cellular environments that can be evaluated in real time, easily scaled and replicated, allowing the evaluation of the effects of drugs, chemical compounds, exhausts or NPs on lung tissues, and reducing the use of animal models and clinical studies. Leveraging these models could aid the discovery of novel therapeutic targets, may provide powerful, scalable screening platforms to test the effects of pharmaceuticals, and can act as an important preclinical step to bridge the gap between drug testing in animal models - which are expensive and have a high failure rate - and human clinical trials.^10^ In fact, several advanced in vitro systems have been recently used to model pathological conditions,^11^ revealing that, in some cases, they are able to perform a match comparison between the responses from normal cells and disease-exposed cells from the same patient, which is an important step toward personalized medical therapy.^12^

The term “physiologically relevant” is often used in the context of in vitro models and is referred to the likeness of the model with respect to the in vivo counterpart. Considering the microenvironment of the alveolus, which is the functional unit of the lung where gas exchange and particles absorption take place, the specifications for an ideal “physiologically relevant” engineered human in vitro model are:

- Human-derived cells that compose the native alveolar barrier (thickness ≈ 0.6 µm,^13^ alveolar surface area ≈ 130 m^2^),^14^ consisting ideally of: an epithelial layer of simple squamous epithelium (i.e. pneumocytes and macrophages); a layer of endothelial cells of the capillary wall; and the basement membrane between the two. Lung cells must be cultured using defined protocols, without losing their phenotypic characteristics;
- A fluidic system that reproduces the blood flow through the alveolar capillaries (mean velocity ≈ 1 mm/s, flow rate 2–5 mL/min in an adult)^15,16^ and provides adequate oxygenation and nutrients to the cell, as well as physiological shear stress to endothelial cells (around 1.5 Pa)^17^;
- An air-liquid interface (ALI) that mimics in vivo microenvironment where the epithelial lung cells are in contact with humid air on one side (which may contain particulate matter in the form of droplets or aerosols) and blood on the other;
- A substrate for growing cells with properties similar to native tissue in terms of chemical composition and biomechanical behavior. Moreover, to reproduce alveolar barrier motion during breathing, this substrate must be subjected to mechanical cyclic stretching (around 5%–12% strains at 0.2 Hz in physiological conditions, and up to 20% strain in some pathological conditions).^18^

**Figure 1.**
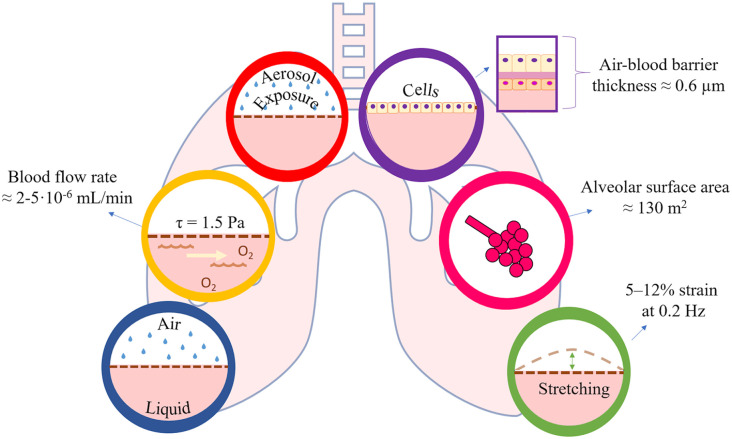
Toward physiological relevance—main elements of the lung microenvironment that are desirable in an in vitro model.

To be physiologically relevant, an in vitro model should replicate as many as possible of the essential features of the tissue or organ it is intended to represent, and which are fundamental for the experimental endpoints that must be evaluated. Thus, alveolar models can be designed with some or all of these requirements (summarized in Figure 1) according to the type of study under consideration and the specific questions being addressed (e.g. a model used to study differentiation or inflammation will have different requirements for physiological relevance than a model used for toxicology or safety applications).

The most important determinant of any in vitro model is the biological component, that is, the cells. Cells for in vitro models of the lung have been amply discussed in some excellent reviews and the reader is encouraged to refer to these for more in depth biological information and comparisons.^19,20^ Generally, they can be obtained from donors, primary cells, cell lines, or human pluripotent stem cells (hPSCs). Commonly employed human epithelial cell lines are the A549 and NCI-H441, for the assessment of the alveolar barrier, and the Calu-3, BEAS-2B, and 16HBE for the assessment of the bronchial barrier.^2^ A promising alternative to cell lines are the hPSCs, which open the possibility to develop patient-specific models.^19^ hPSCs could indeed win the debate over the ideal cell source, but currently the need for protocol optimization and standardization is still an obstacle. Additionally, lung epithelial cells can be co-cultured with vascular, neural, or immune cells such as macrophages, dendritic cells, and mast cells.^1,2,19–23^ The co-cultures enhance the reliability of the in vitro lung model, making them more similar to the complex in vivo microenvironment. Regardless of the type of cells, there seems to be an agreement among the scientific community: models in which cells are cultured at the *air-liquid interface (ALI)* better represent the physiological environment of the lung. Indeed, a wide variety of studies have been performed comparing the culture of lung cells in ALI and in submerged conditions, revealing that cells displayed phenotypic differences.^20^

Besides the cells, the choice of the most appropriate experimental setup is crucial for the design of an ad hoc in vitro model. A variety of models have been proposed using different engineering solutions that we will discuss in the next sections of this review. To facilitate the analysis of the devices, we have grouped them in:

- Fluidic systems that provide adequate oxygenation and nutrients to the cells, as well as physiological shear stress.
- Systems that combine ALI culture with direct and quantitative aerosol/smoke exposure, for toxicological studies and drug testing.
- Devices that mimic mechanical stretch of lung tissues during breathing.
- Lungs-on-chips, which mimic biological and/or biochemical processes at the micro-scale.

In each section we analyze the biological role of the mechanical stimuli and exposure on lung tissues and then critically assess the approaches that have been employed to recreate such dynamic conditions.

## Dynamic lung models: Fluidic systems

Shear stress is the frictional force per unit surface area exerted at a fluid-solid interface when they are in relative motion. The vascular system of the lung is continuously exposed to shear stress from blood flow. Furthermore, shear stress is also generated on the gas side from the airflow exerted on the airways.^2^ It has been demonstrated that shear stress modulates different cellular phenomena such as morphology, proliferation, differentiation, metabolism, and communication.^24^

**Figure 2.**
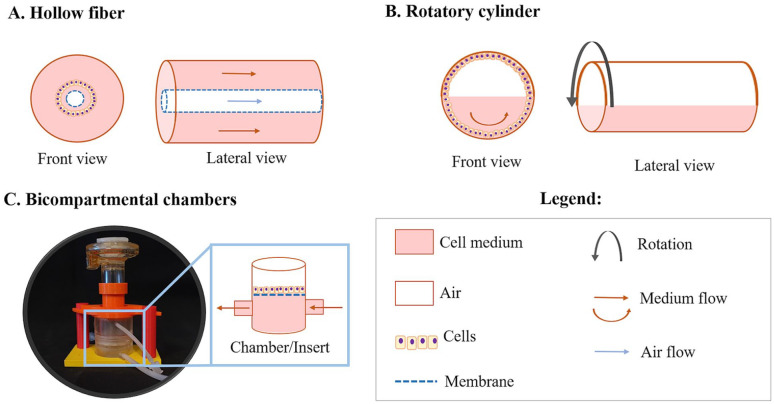
Different configurations of bioreactors designed to operate with millifluidics. Representations are not at scale. The photograph in panel C shows the MALI chamber with nebulizer.^158^

Several research groups have therefore developed dynamic systems that are able to provide shear stress while enhancing oxygen and nutrient diffusion at the same time. A variety of solutions have been adopted to generate cell cultures with medium flow using bioreactors with different configurations, as schematized in Figure 2.

Hollow fiber bioreactors, for example, are common tools for performing dynamic cultures of many tissues^25^ and the lung is no exception.^26^ Here, air and cell culture media flow through the system through appropriate connectors providing the ability to modulate the environment both in the lumen of and surrounding the semipermeable fibers. An interesting feature of this type of system while comparing with other solutions, is that the cell culture can experience a nearly physiological air and fluid flow environment. Unlike cells grown in conventional 2D static culture systems, cells grown in these bioreactors show typical characteristics of differentiation.^26^ Another way of providing both ALI and adequate shear stress to the cells is by culturing them in rolling bioreactors.^27^ As the system rolls, the cells spend an equal amount of time in air and liquid, rendering this bioreactor a suitable tool to study the impact of ALI on the cell differentiation process. However, unlike hollow fiber-based systems, it does not faithfully represent the in vivo configuration. Finally, a prevalent choice for the provision of medium flow is through the use of bioreactors with a tubing system and pumps.^28–34^ In these models, well inserts are typically cultured with epithelial and endothelial cells, on the apical and basal sides, respectively. The endothelial compartment is connected to the tubing system and thus sustained by media flow, which can be controlled to regulate the level of shear stress on the cells. Exploring this approach, dynamic in vitro models of invasive pulmonary aspergillosis were set up to study the pharmacodynamics of voriconazole^32^ and isavuconazole.^31^ Toxicology studies have also been performed to evaluate the effects of NPs (e.g. gold NPs),^34^ or other airborne materials such as pollen.^33^ These examples illustrate that the use of flow systems is relevant for studies, where the role of shear stress on cell responses is assessed, and for absorption studies, where more complex kinetics and dynamics are considered. The characteristics of the systems employed to perform these studies are summarized in Table 1.

**Table 1.** Specifications of fluidic systems developed for lung in vitro studies (n.m.: not mentioned). Values were compiled as published in the original sources, RWV: Synthecon Rotating Wall Vessel.

Authors	Type of bioreactor	Medium volume	Cell type	Cell density	Flow rate	Shear stress	Study
Grek et al.^26^	Hollow fiber	n.m.	MLE-15	8.6 × 10^4^ cells/cm^2^	Air flow: 1 × 10^−2^ mL/min; medium flow: n.m.	n.m.	Assessment of phenotypic characteristics
Ghaedi et al.^27^	Rotating cylinder	15 mL	iPSC-ATII and hATII	2 × 10^5^ cells/cm^2^	n.m.	n.m.	Stem cell differentiation protocols
Jeans et al.^32^	Inserts housed in bioreactors	200 mL	Co-culture of HPAECs and A549s	1 × 10^6^ cells/cm^2^ and 5.5 × 10^5^ cells/cm^2^ (respectively)	0.17 mL/min	n.m.	Human-like voriconazole pharmacokinetics
Box et al.^31^	Inserts housed in bioreactors	n.m.	Co-culture of HPAECs and A549s	1 × 10^6^ cells/cm^2^ and 5.5 × 10^5^ cells/cm^2^ (respectively)	0.17 mL/min	n.m.	Human-like isavuconazole pharmacokinetics
Blume et al.^33^	Inserts housed in microfluidic circuit	n.m.	PBECs	n.m.	5 × 10^−4^ mL/min	n.m.	Biological responses to pollen exposure
Breitner et al.^34^	Multiwell plate connected to pump	n.m.	A549s	8.4 × 10^4^ cells/cm^2^	7.5 × 10^−1^ mL/min;	n.m.	Nanoparticle evaluation
Aufderheide et al.^35^	Cultex LTC-C	35 mL	NHBE048	1–1.5 × 10^5^ cells/cm^2^	Rotation speed: 3 rpm	n.m.	Cultivation of airway epithelial cells at ALI
Carterson et al.^36^	RWV bioreactor	n.m.	A549s	5 × 10^6^ cells in microcarrier beads	n.m.	Note: The bioreactor delivers the same terminal velocity and consequently shear stress to similar-sized particles, independent of the rotation speed.	Interactions between Pseudomonas aeruginosa and lung epithelial cells
Cortiella et al.^37^	RWV bioreactor	n.m.	mESCs	2 × 10^6^ cells/construct	2 rpm	Note: The bioreactor delivers the same terminal velocity and consequently shear stress to similar-sized particles, independent of the rotation speed.	Acellular lung as a matrix to develop an engineered lung tissue
Crabbé et al.^38^	RWV bioreactor	50 mL	MSCs and C10	4 × 10^6^ cells/scaffold (MSCs/C10)	20 rpm	Note: The bioreactor delivers the same terminal velocity and consequently shear stress to similar-sized particles, independent of the rotation speed.	Enhance the cell repopulation of decellularized lungs
Wilkinson et al.^39^	RWV bioreactor	n.m.	Lung organoids: FLFs, HUVECs, and SAECs; or iPSCs	1 mL alginate beads + 4 × 10^6^ FLFs/iPSC; 100 μL alginate beads + 1.5 × 10^5^ SAECs	4–30 rpm	Note: The bioreactor delivers the same terminal velocity and consequently shear stress to similar-sized particles, independent of the rotation speed.	Generation of self-assembled human lung tissue
Crabbé et al.^40^	RWV bioreactor	n.m.	A549s	5 × 10^6^ cells in microcarrier beads	n.m.	Note: The bioreactor delivers the same terminal velocity and consequently shear stress to similar-sized particles, independent of the rotation speed.	Pseudomonas aeruginosa biofilm susceptibility on biotic surfaces

Since the culture of cells in dynamic conditions became popular, optimized commercial solutions have been developed based on the operating principles represented in Figure 2. Among the commercial devices, lung models using Synthecon (Synthecon^®^ Incorporated, Houston, Texas, USA) and Cultex LTC-C (Cultex^®^ Technology, Hannover, Germany) have been widely reported. Synthecon systems are perfusion bioreactors that comprise a cylindrically shaped rotating vessel with a central gas transfer core, while the Cultex LTC-C system presents a hydraulic circuit with media flow facilitated by peristaltic pumps. The main application of the latter is the generation of comparable cultures for mechanistic and toxicological studies.^41^ On the other hand, Synthecon bioreactors are better tailored for cell/tissue engineering approaches. They have mainly been used to produce whole acellular lung as a matrix to support the development of engineered lung tissue,^37,38^ to evaluate antimicrobial efficacy against biofilm formation in 3D lung epithelial models,^40^ and for the generation of self-assembled human lung tissue (organoids)^39^ employed for disease modeling and drug discovery. The employment of more sophisticated fluidic devices is analyzed in the following section and they are an option for researchers looking for further complex systems compatible with exposure studies.

From Table 1, it is clear that the protocols are not universal for all the lung models cultured in dynamic conditions. Besides varying in their working principle, they also vary in scale: the “in-house” fluidic circuits go from micro to milli-scale and even commercial devices, such as the ones from Synthecon, can work with a wide range of volumes (from 1 to 50 mL for one of the available configurations, according to their website). Regarding the biological components, both cell density and cell medium flow rate vary greatly. For the models with cells cultured in 2D, the epithelial cell densities range from 8000 to 500,000 cells/cm^2^, but this range expands as the cellular complexity of the model increases (both in composition and in arrangement). When it comes to the flow rate, the data is not always available and in a number of cases it was derived from descriptions of the fluidic circuit set-ups. Remarkably, the flow rates are considerably higher than the pulmonary capillary flow rate of ≈2–5 10^−6^ mL/min in a human.^15^ Only the device developed by Blume et al.^33^ applied a medium flow (5 × 10^−4^ mL/min) that was relatively close to the physiological range. The shear stress provided by all these systems is often referred to as “low-shear stress” but the values are not estimated/presented. Clearly there is a need to harmonize reporting to enable more precise identification and implementation of the flow-related parameters in the studies. Only then can different approaches be compared, and meaningful correlations be identified between cellular response and a certain stimulus or type/magnitude of stimulus. Nonetheless, these bioreactor systems are a step forward in the design of advanced in vitro models, when compared with more traditional systems.

## Knowing what we breathe: Combining ALI culture and aerosol/smoke exposure

Inhalation is an important route of exposure to particulates, both in the form of drugs if considering pharmaceutical therapies, and environmental particles.^42^ The goal of pharmaceutical therapies is to allow drug delivery into the lung with maximum efficiency, while the effect of environmental particles must be investigated to evaluate their potential toxicity on lung tissues. Therefore, several studies are focused on assessing the biopharmaceutics and toxicology following particulate exposure, and several models have been developed to investigate these aspects. Progress in this direction is represented by complex in vitro models that combine ALI culture and aerosol/smoke exposure making them suitable for studies on inhalation toxicology and pharmacology. The following subsections present both laboratory-made and commercial systems that belong to this category.

### Laboratory-made systems for aerosol exposure

Over the years, several authors have focused on the design and characterization of innovative systems in terms of deposition efficiency and homogeneity, to maximize experimental reliability and throughput. Indeed, one of the essential requirements for exposure systems is the ability to present compounds or materials directly and reproducibly to cells in culture so as to allow dose-response analyses of airborne molecules or materials. They have been used principally to investigate the inflammatory and toxic effects of aerosolized compounds on lung tissue, combining ALI culture with direct exposure of gaseous contaminants (i.e. NO_2_ and O_3_),^43,44^ volatile organic compounds,^45^ brake powder,^46^ diesel exhaust particles,^47–50^ or micro/nanoparticles.^51–53^ The exposure is performed using different approaches: with gas generators,^43,44^ commercial microsprayers,^51^ flame spray synthesis,^54^ or specifically designed solutions. For example, Riediker et al. worked on a device that consisted in an exposure-box mounted around a car’s braking system to collect, purify, and nebulize brake powders;^46^ Cooney,^47^ Holder,^48,49^ and Oosting^50^ designed custom exposure systems and deposition chambers to evaluate the effect of diesel exhaust particles on lung cells cultured on Transwell inserts; another even simpler approach was proposed by Bakand et al., who placed cells cultured at ALI in a glass chamber at 37°C, together with filter paper soaked with volatile compounds (i.e. Toluene and Xylene).^45^

Other studies focused on the quantitative characterization of therapeutic aerosols in vitro, using modified pharmaceutical impactors and impingers, which operate on the principle of inertial impaction. These devices consist in a series of stages with a single or multiple nozzles or jets through which the aerosol flow is driven. If particles have sufficient inertia, they will impact on that particular stage collection plate; if not, they will remain entrained in the air stream and pass to the next stage where the process is repeated. This allows characterizing the particle size distribution of aerosols. The term “impactor” is generally used when the particles impact on a dry impaction plate or cup, while “impinger” refers to a liquid collection surface.^55^ For the aerodynamic assessment of fine particles, the European Pharmacopoeia recommends: the twin stage impinger (TSI), multi stage liquid impinger (MSLI), next generation impactor (NGI), and Andersen cascade impactor (ACI).^55^ Even though these tools are useful for evaluating the aerodynamic performance of aerosol formulations, they do not give information relating to drug dissolution and transport at the epithelia. Therefore, in order to study the deposition and transport of inhaled drugs across the epithelial barrier, several researchers modified these impactors inhouse incorporating *in vitro* cell based methods into classical impactors to provide a better understanding of the fate of microparticles after deposition in the respiratory tract.^56–59^

Another approach used to design predictive lung in vitro models consists in the introduction of media flow combined with exposure at ALI. This setup better reproduces the alveolar microenvironment, where the blood flow through the capillaries is reproduced by the media flowing through the “liquid side” of the ALI interface. The media flow also enhances oxygen and nutrient diffusion and provides shear stress to the cell surface. In this context, Tippe et al. modified the commercially available perfusion Minucell device (MINUCELL, D-93077 Bad Abbach, Germany) to evaluate the quantitative dosimetry of fine and ultrafine aerosol particles during in vitro exposure and permit an aerosol exposure by stagnation point flow.^60^ Successively, other authors used this approach to allow a dose-controlled exposure of ultrafine- and nano-particles.^61,62^

Finally, several studies show that applying an electric field during exposure leads to better control of particle precipitation, enhancing the deposition efficiency, reproducibility, and uniformity of particles on the cell culture surface. Therefore, electrode-assisted systems were used to evaluate the deposition and electrical discharge on cell layers during aerosolization, analyzing the toxicity of nebulized micro-^63^ and nano-particles,^42,52,53,64^ diesel exhaust,^63,65,66^ or air pollutants.^67^

### Commercial aerosol and smoke exposure devices

The most commonly used commercial systems for the direct and quantitative exposure to aerosols are the Cultex^®^ RFS system, the Vitrocell^®^ exposure chambers, and the PreciseInhale. In these systems the aerosol generator is connected to an exposure chamber in which the well inserts (or Petri dishes) are placed, and the cell culture media is supplied individually to each well compartment, ensuring the ALI. Although Vitrocell systems are designed to perform ALI culture, they have been used to test both submerged and ALI experimental conditions, investigating the effects of each exposure scenario.^68–71^

The successful application of these commercial systems is demonstrated by the high number of toxicological testing studies to which they are applied. They can also be used to evaluate the therapeutic potential of new formulations. For instance, Lenz et al. investigated the effect of a commercial FDA-approved proteasome inhibitor (Bortezomib).^72^ Another example is Schmid et als.’ study of the biokinetic behavior of the immunosuppressive drug Cyclosporin A encapsulated in liposomes at the lung epithelial barrier.^4^ Still in the field of drug testing, Gerde et al. and Malmlöf et al. used the PreciseInhale to evaluate dissolution and adsorption in the lungs of drugs such as Fluticasone propionate,^73,74^ Budesonide,^73,74^ and Salmetrol.^74^ Tables 2 and 3 summarize the classes of substances and testing conditions using these systems, splitting them respectively into chemical/biological compounds and nanomaterials.

**Table 2.** Chemical compounds aerosolized using the Cultex and Vitrocell exposure chambers.

Tested substances		Authors	System used	Cell type	Cell density	Compound concentration	Aerosolization mode
**Biological compounds**	*Aspergillus fumigatus*	Persoz et al.^75^	Vitrocell	A549	1.8·10^4^/cm^2^	7·10^8^ spores/m^3^	*T*_exp_: 30 min; *Q*: 5 ± 0.1 mL/min
**Organic compounds**	Benzene	Pariselli et al.^76^	Cultex	A549	1.5·10^4^/cm^2^	0.28 ± 0.03 ppmv	*T*_exp_: 1 h; *Q*: 2 mL/min
	Dicarbonyls	Anderson et al.^77^	Vitrocell	A549	53 533/cm^2^	15–65 ppm	*T*_exp_: 2/4 h; Flow rate: 3 mL/min
	Formaldehyde	Persoz et al.^75^	Vitrocell	A549	1.8·10^4^/cm^2^	50 µg/m^3^	*T*_exp_: 30 min; *Q*: 5 ± 0.1 mL/min
		Bardet et al.^78^	Vitrocell	hAECN	1·10^5^/cm^2^	200 µg/m^3^	*T*_exp_: 1 h for 1, 2, 3 times at 24-h intervals *Q*: 2 ± 0.1 mL/min
	Toluene	Pariselli et al.^76^	Cultex	A549	1.5·10^4^/cm^2^	0.25 ± 0.06 ppmv	*T*_exp_: 1 h; *Q*: 2 mL/min
		Al Zallouha et al.^79^	Vitrocell	A549	1.1·10^5^/cm^2^	100 and 1000 ppm	*T*_exp_: 1 h *Q*: 100 mL/min
		Méausoone et al.^80^	Vitrocell	BEAS-2B	1500/cm^2^	100 and 1000 ppm	*T*_exp_: 1 h over 5 days; Q: 100 mL/min
	Dihydroxyacetone (DHP)	Wang et al.^81^	Vitrocell	NHTBE	Suspension of 4·10^5^ cells/mL 100 µL added to a 24-well Transwell insert	DHP dissolved in DPBS to 0.2, 0.4, and 1 M	*T*_exp_: 30 s; *Q*: n.m.
	Phthalic anhydride	Chary et al.^82^	Vitrocell	A549+THP-1+EA.hy 926	EA.hy 926: 2.4·10^4^/cm^2^ A549: 6·10^4^/cm^2^ THP-1: 2.4·10^4^/cm^2^	Stock solutions diluted in 50% (v/v) sterile water in PBS 1X	*T*_exp_: 15 min; *Q*: n.m.
	Trimellitic anhydride						
	Methyl salicylate						
	Acrolein						
	Limonene	Anderson et al.^83^	Vitrocell	A549	2.8·10^4^–1.1·10^5^/cm^2^	20 ppm	*T*_exp_: 1–4 h; *Q*: 3 mL/min
				MucilAir	n.m.	500 ppb	*T*_exp_: 1 h per day, 5 days per week/4 weeks *Q*: 2 mL/min
**Inorganic compounds**	Ozone	Anderson et al.^83^	Vitrocell	A549	2.8·10^4^–1.1·10^5^/cm^2^	4 ppm	*T*_exp_: 1–4 h; *Q*: 3 mL/min
				MucilAir	n.m.	100 ppb	*T*_exp_: 1 h per day, 5 days per week/4 weeks *Q*: 2 mL/min
	Phosgene	Olivera et al.^84^	Vitrocell	16HBE	5.8·10^5^/cm^2^	1, 2, 4, 8, 16, 32, and 64 ppm	*T*_exp_: 8 min; *Q*: 8.3 mL/min
	Copper(II) oxide micro	Aufderheide et al.^85^	Cultex	A549	1·10^5^/cm^2^	n.m.	*T*_exp_: 15/30/60 min; *Q*: 30 mL/min
**Complex mixtures**	Cigarette smoke	Okuwa et al.^86^	Cultex	Chinese hamster lung cells	1.1·10^5^/cm^2^	n.m.	According to ISO 3308 (35 mL puff volume, 2 s duration, 1 puff/min) *T*_exp_: 4 h; *Q*: 5 mL/min
		Aufderheide et al.^87^	Cultex	16HBE14o^-^	n.m.		
		Nara et al.^88^	Cultex	CHO-K1	4.4–5.6·10^4^/cm^2^		
		Rach et al.^89^	Cultex	16HBE14o^-^	2.5·10^5^/cm^2^		
		Aufderheide et al.^40^	Cultex	NHBE	1–1.5·10^5^/cm^2^		
		Scheffler et al.^90^	Cultex	NHBE+A549+CL 1548	NHBE: 2.1·10^5^/cm^2^ A549: 2.5·10^5^/cm^2^ CL 1548: 2.5·10^5^/cm^2^		
		Scheffler et al.^91^	Cultex	NHBE	2.1·10^5^/cm^2^		
	E-liquid aerosol	Scheffler et al.^90^	Cultex	NHBE+A549+CL 1548	NHBE: 2.1·10^5^/cm^2^ A549: 2.5·10^5^/cm^2^ CL 1548: 2.5·10^5^/cm^2^		
		Scheffler et al.^91^	Cultex	NHBE	2.1·10^5^/cm^2^		
	Exhaust fumes (HFO: Heavy Fuel Oil; DF: Diesel Fuel, DE: Diesel Exhaust, DEPM: Diesel Exhaust Particulate Matter)	Oeder et al.^92^	Vitrocell	A549/BEAS-2B	8.9·10^4^/cm^2^	DF: 28 ± 1.5 µg/cm^3^ HFO: 56 ± 0.7 µg/cm^3^	According to ISO 8178-4 E2 *T*_exp_: 4 h
		Sapcariu et al.^93^	Vitrocell	RAW 264.7	2.1·10^5^/cm^2^	DF: 340 µg/cm^3^ HFO: 760 µg/cm^3^	
		Klein et al.^94^	Vitrocell	A549 + THP-1 + EA.hy 926 + HMC1	EA.hy 926: 2.4·10^5^/cm^2^ A549: 1.2·10^5^/cm^2^ THP-1: 2.4·10^5^/cm^2^ HMC1: 1.2·10^5^/cm^2^	Dose of DEPM: 40, 80 and 240 ng/cm^2^	*T*_exp_: 1 min 8 s (40 ng/cm^2^) 2 min 17 s (80 ng/cm^2^) 6 min 52 s (240 ng/cm^2^) *Q*: 5 ± 0.1 mL/min
		Kooter et al.^95^	Vitrocell	A549	~0.1·10^5^/cm^2^	n.m.	DE exposure according to the European Commission directive 2005/78/EC T_exp_: 1.5 h;
		Tsukue et al.^96^	Cultex	A549	1.67·106/cm2	DEPM: 0.07–0.85 mg/m3 Gaseous components: 0.2–45.8 ppm	*T*_exp_: 1 h; *Q*: 8.3 cc/min/insert
		Ji et al.^97^	PreciseInhale	PBEQ + MQ	PBEQ: 1·10^5^/cm^2^ MQ: 5.6·10^5^/cm^2^	Dose of DEPM: 1.7 µg/cm^2^	*T*_exp_: 3 min; *Q*: 10 mL/min
	Emission from laser printers	Tang et al.^98^	Vitrocell	A549	n.m.	n.m.	*T*_exp_: 1 h; *Q*: 5 mL/min
	Smoke particles emitted by a household log wood stove	Mülhopt et al.^99^	Vitrocell	A549	8.6·10^4^/cm^2^	Particle density in wood exhaust: 2.7 g/cm^3^	Stove fired according to DIN EN ISO 17225-5 *T*_exp_: 4 h; *Q*: 100 mL/min
	Fly ash collected from a municipal waste incinerator	Diabaté et al.^100^	Cultex	BEAS-2B THP-1	BEAS-2B: 1.1·105/cm2	~3.6·104 particles/cm^3^	*T*_exp_: 1 h; *Q*: 300 mL/min
	Indoor gaseous pollutants	Bardet et al.^78^	Vitrocell	hAECN	1·10^5^/cm^2^	n.m.	*T*_exp_: 1 h for 1, 2, 3 times at 24-h intervals *Q*: 2 ± 0.1 mL/min

n. m.: not mentioned; Q: flow rate; T_exp_: exposure time.

**Table 3.** Aerosolized nanoparticles using the Cultex and Vitrocell exposure chambers.

Tested NP/NT	Authors	System used	Primary NP/NT diameter	NP/NT concentration	Aerosolization mode	Cell type	Cell density
**Zinc oxide**	Xie et al.^68^	Vitrocell	25 nm	NPs suspended in sterile water (5 mg/mL) and diluted 1-, 2-, 10-, 50-, and 100-fold	*T*_exp_: 10–20 min *Q*: 10 mL/min	C10	n.m.
	Stoehr et al.^71^	Vitrocell	35 nm	0.5 and 4.25 mg/mL in MilliQ dH_2_O	*T*_exp_: 15 min *Q*: n.m.	A549	2.4·10^4^/cm^2^
	Mihai et al.^101^	Vitrocell	25 nm	NP solution diluted at 0, 0.05, 0.20, 0.30, 0.50, 1.00, and 1.50 mg/mL	*T*_exp_: 10 min *Q*: 10 mL/min	C10	2.4·10^4^/cm^2^
**Silica**	Klein et al.^102^	Vitrocell	50 nm	1 g/L in PBS	*T*_exp_: 30 min *Q*: 5 mL/min	A549 THP-1 EA.hy 926 HMC-1	A549: 1.2·10^5^/cm^2^ THP-1: 2.4·10^5^/cm^2^ EA.hy 926: 2.4·10^5^/cm^2^ HMC-1: 1.2·10^5^/cm^2^
	Panas et al.^103^	Vitrocell	50 nm	1, 3.25 and 7 mg/mL in dH_2_O	*T*_exp_: 5 or 7 h *Q*: 100 mL/min	A549	8.5·10^4^/cm^2^
**Pristine and carboxylated copper oxide NPs**	Kooter et al.^104^	Vitrocell	10–20 nm	6.15·10^5^ and 1.65·10^6^ particles/cm^3^	*T*_exp_: 1 h *Q*: 1.5 mL/min	MucilAir	n.m.
**Hybrid lipid-polymer**	D’Angelo et al.^105^	Vitrocell	~150 nm	0.5 and 0.9 mg/mL	*T*_exp_: 10 and 30 min *Q*: 15 L/min	16HBE14o^-^ MDM MDDC	16HBE14o^-^ : 0.55·10^6^/cm^2^ MDM: 2.5·10^4^/mL MDDC: 83·10^4^/mL
**Cerium oxide**	Steinritz et al.^106^	Cultex	15–30 nm	Concentration of 25 µg/cm^2^ deposited mass within 15 min exposure	*T*_exp_: 15, 30 and 60 min *Q*: 1.5 L/min	A549	1·10^5^/cm^2^
	Rach et al.^89^	Cultex	50–80 nm	Concentration of 25 µg/cm^2^ deposited mass within 15-min exposure	*T*_exp_: 15–60 min *Q*: 5 mL/min	A549	1·10^5^/cm^2^
	Kooter et al.^107^	Vitrocell	13.8 and 750 nm	50 mg/m^3^	*T*_exp_: 1 h *Q*: 1.5 and 5 mL/min	A549 BEAS-2B MucilAir	A459 and BEAS-2B: 9524/cm^2^ MucilAir: NA
	Loret et al.^69^	Vitrocell	29 nm	7.9–105.7 mg/m^3^	*T*_exp_: 3 h *Q*: 5 mL/min	A549 THP-1	A549: 17,130/cm^2^ THP-1: 1713/cm^2^
	Cappellini et al.^108^	PreciseInhale	~30 nm	2.56 mg/mL in BSA	*T*_exp_: 20 min *Q*: 5 mL/min	A549 THP-1	A549: 30,000/cm^2^ THP-1: 46,666/cm^2^
**Titanium dioxide**	Loret et al.^69^	Vitrocell	8, 21 and 100 nm	10.6–113.5 mg/m^3^	*T*_exp_: 3 h *Q*: 5 mL/min	A549 THP-1	A549: 17,130/cm^2^ THP-1: 1713/cm^2^
	Rach et al.^89^	Cultex	25 nm	Concentration of 25 µg/cm^2^ deposited mass within 15-min exposure	*T*_exp_: 15–60 min *Q*: 5 mL/min	A549	1·10^5^/cm^2^
	Steinritz et al.^106^	Cultex	25 nm	Concentration of 25 µg/cm^2^ deposited mass within 15-min exposure	*T*_exp_: 15, 30 and 60 min *Q*: 1.5 L/min	A549	1·10^5^/cm^2^
**Carbon black**	Steinritz et al.^106^	Cultex	14 nm				
**Magnesium oxide**	Steinritz et al.^106^	Cultex	n.m.				
**Barium sulfate**	Steinritz et al.^106^	Cultex	n.m.				
**Copper(II) oxide**	Steinritz et al.^106^	Cultex	40 nm				
	Aufderheide et al.^85^	Cultex	40–80 nm	n.m.	*T*_exp_: 15 and 60 min Deposition rate: 25 µg/cm^2^/15 min	A549	1·10^5^/cm^2^
**Copper**	Kim et al.^109^	Vitrocell	25 nm	1 mg/mL	*T*_exp_: NA *Q*: 5 mL/min	A549	1.7·10^5^/cm^2^
	Elihn et al.^110^	Cultex	180 ± 1.5 nm	105 particles/mL	*T*_exp_: 4 h (constant and pulsed aerosol flow) *Q*: 20 mL/min	A549	0.43·10^5^/cm^2^
**Gold**	Bachler et al.^111^	Vitrocell	2, 7, 18, 46 and 80 nm	170, 200, 300, 200, and 220 µg/mL	*T*_exp_: 15 min *Q*: 5 mL/min	A549 16HBE14o^-^ MLE-12	0.56·10^6^/cm^2^
	Durantie et al.^112^	Vitrocell	~ 32 nm	0.05, 0.1, 0.25, and 0.5 mg/mL	*T*_exp_: 10 min *Q*: n.m.	A549 MDM MDDC	A549: 120·10^4^/cm^2^ MDM: 5.56·10^4^/cm^2^ MDDC: 1436·10^4^/cm^2^
	Chortarea et al.^113^	Vitrocell	~58 nm	120 µg/mL	*T*_exp_: n.m. *Q*: 5 L/min	A549 MDM MDDC	A549: 9714/mm^2^ MDM: 411/mm^2^ MDDC: 231/mm^2^
**Multi-walled carbon NTs**	Chortarea et al.^114^	Vitrocell	n.m.	25, 125, and 250 µg/mL in Pluronic F127	*T*_exp_: n.m. *Q*: 5 L/min	A549 MDM MDDC	A549: 9714/mm^2^ MDM: 411/mm^2^ MDDC: 231/mm^2^
	Chortarea et al.^115^	Vitrocell	n.m.	250 µg/mL in Pluronic F127	*T*_exp_: n.m. *Q*: 5 L/min	MucilAir	n.m.
	Beyeler et al.^116^	Vitrocell	Length: 2–16 µm Inner diameter: 2–13 nm Outer diameter: 6–34 nm	25 µg/mL in Pluronic F127	*T*_exp_: n.m. *Q*: 5 L/min	Primary bronchial epithelial cells	n.m.
Palladium	Ji et al.^117^	PreciseInhale	6–10 nm	n.m.	*T*_exp_: 20 s, 45 s and 3 min *Q*: 10 mL/min	PBEC MRC-5	PBEC: 1·10^5^/cm^2^ MRC-5: 1·10^4^/cm^2^

n. m.: not mentioned; Q: flow rate.; T_exp_: exposure time.

In addition to the systems for direct aerosol exposure, several commercial smoking machines combined with exposure chambers have been designed particularly for smoke inhalation simulation: examples are the Vitrocell VC smoking machine (Vitrocell^®^ systems, Waldkirch, Germany) and the Borgwaldt systems (Borgwaldt KC, Hamburg, Germany), the latter usually paired with the British American Tobacco (BAT) exposure chamber.^118^

Several studies illustrate the value of these systems, which can be found in the papers by Thorne who provided a comprehensive review of the major tobacco smoke exposure systems available to 2013,^118^ and a comparison of in vitro data across multiple smoke exposure studies using reference cigarettes and considering three different smoking machines.^119^ In the latter review, Thorne demonstrated that in vitro dosimetry techniques can align data between contrasting setups and experimental protocols, resulting in a link between in vitro, in vivo, and human dosimetry studies.

Although smoking machines have been used principally to investigate the effects of cigarette smoke on lung tissues,^120–129^ in the last few years, several research groups have focused their efforts on studying the effects of next generation tobacco and nicotine products, namely e-cigarette aerosols and heated tobacco products.^130–140^

Table 4 shows all the commercial technologies mentioned here, which can be considered a good choice for advanced in vitro models if exposure conditions related with inhalation are a crucial aspect of the study.

**Table 4.** Commercial systems for aerosol and smoke exposure, highlighting their similarities and differences.

	**Aerosol Exposure**						**Smoke exposure**	
**System**	Cultex RFS	Vitrocell			PreciseInhale		Vitrocell VC	Borgwaldt systems
		Exposure chambers	Powder chamber	Cloud system				
**Configuration**	Stand alone	Combined with aerosols generators or gas supply systems	Stand alone	Stand alone	Combined with Xpose*ALI* cell exposure unit	Combined with Dissolv*It* module	Combined with the Vitrocell exposure chambers	Combined with the BAT chamber
**Aerosolized substances**	Airborne substances (gases, NPs, complex mixtures, fibers)	Airborne substances (gases, NPs, complex mixtures, fibers)	Specific for dry powders	Specific for liquid aerosols	Airborne particles	Specific for dry powders	Smoke generation	Smoke generation
**Media flow**	Yes	Yes	Yes	Yes	Yes	Yes	Yes	Yes
**QCM**	No	Yes	Yes	Yes	No	No	No	No

To summarize, all the technologies mentioned above have somehow increased the complexity of lung models compared to traditional static culture. In these studies, the goal was to investigate the effect of aerosol/smoke deposition on lung tissues; fundamental to this scope is culturing cells at the ALI, to reproduce the in vivo deposition of the inhaled particulate. Some researchers devised very simple but functional solutions for their purposes (i.e. Bakand et al.),^45^ while others increased the model complexity by introducing culture medium flow, or performing electrical deposition. Finally, some authors modified pharmaceutical impactors, already used to characterize the particle size distribution of pharmaceutical aerosols, to obtain ad hoc in vitro models and study the deposition and transport of inhaled drugs. In addition to these laboratory-made devices, a large number of investigations are conducted using commercial systems, which at least allows some comparisons to be made between data from different studies. However, we should underline that although there are many reports on exposure systems, very little attention is paid to precise measures of dosimetry, which greatly reduces the strength of comparative analyses. (Source PubMed—from 2010 to 2020: 55 documents on inhalation exposure and dosimetry in vitro and 1076 on inhalation exposure in vitro). In general, commercial systems are always the best choice for obtaining comparable results within the scientific community. This is the reason why Cultex and Vitrocell systems have become so diffuse in aerosol deposition studies over the last years. On the other hand, for the quantitative characterization of therapeutic aerosols in vitro, modified pharmaceutical impactors and impingers can be a good alternative. Indeed, these devices recommended by the European Pharmacopoeia are specifically designed for evaluating the aerodynamic performance of aerosol formulations, and, when modified incorporating *in vitro* cell based methods, also give information relating to drug dissolution and transport at the epithelia. However, there are studies in which commercial systems or modified commercial systems are not appropriate, since they do not allow to reproduce/evaluate some elements. For example, in order to control particle precipitation and enhance the deposition efficiency, electrode-assisted systems are the best option. However, to our knowledge there are no commercial in vitro lung models able to apply an electric field during exposure, and for this reason several authors designed ad hoc devices to be used only with charged or chargeable particles. Finally, it should be noted that none of these devices (commercial or not) are able to reproduce the effect of the deformation of lung tissue which occurs during breathing, which may be a crucial modulator of the interaction between the alveoli and inhaled materials as discussed in the following section.

## There is more to breathing than air exchange: Mimicking mechanical stretching

Over the last two decades it has become clear that mechanical stress and deformation influence the biological function and signaling of alveolar epithelial cells.^141,142^ For example, mechanical stretch of cultured alveolar type II cells leads to changes in surfactant secretion,^143–145^ cell injury or death,^143,146–148^ permeability,^149–152^ and cell migration.^153^ However, there are still many unanswered questions regarding the micromechanics of the alveoli and the way in which it affects the mechanisms involved in lung physiology.^141,154^ To better understand the effects of mechanical stimuli on lung epithelia by reproducing breathing motions, several research groups have developed systems able to apply cyclic stretch to cell culture supports.

Most of the currently available in vitro cell-stretching devices are covered in an excellent and systematic review by Doryab et al. that elucidates the relevance of cyclic mechanical forces in lung biology.^155^

In this section, we first discuss and analyze the motion and resulting strains in the alveolus and lung. We then describe milli and micrometer-sized stretching devices, outlining the combined effect of device dimensions, deformation mechanism and stretching directions, and their physiological relevance.

### A brief description of the motion

During spontaneous breathing or mechanical ventilation, pulmonary tissues are permanently subjected to cyclic stretch with varying breathing frequency and volume amplitude in order to pair up with the metabolic state of the subject. In the resting state, the lungs expand and recoil with a frequency of about 0.2 Hz (12 cycles/min) and a tidal volume of around 10% of total lung capacity.^18^

At the macroscale, breathing movements are mainly related to the transpulmonary pressure (i.e. the difference between air pressure in the airways and the pressure at the pleural surface), elastic recoil (related to the high elastin content), and to muscular movements caused by the diaphragm and intercostal muscles. These forces are transmitted at the microscale thanks to the extracellular matrix network, causing linear strain (defined as the variation in alveolar radius with respect to the initial radius) between 4% and 12%.^18^ However, strain levels can increase or decrease in an injured or damaged lung, due to changes in the structure and mechanical properties.

Ex-vivo and computational studies have shown that different deformation mechanisms occur as a function of pressure-volume variations including: (i) recruitment/derecruitment of alveolar units; (ii) folding/unfolding of alveolar walls; (iii) change in alveolar shape (dodecahedral/spherical); (iv) isotropic stretching/destretching.^154,156^

**Figure 3.**
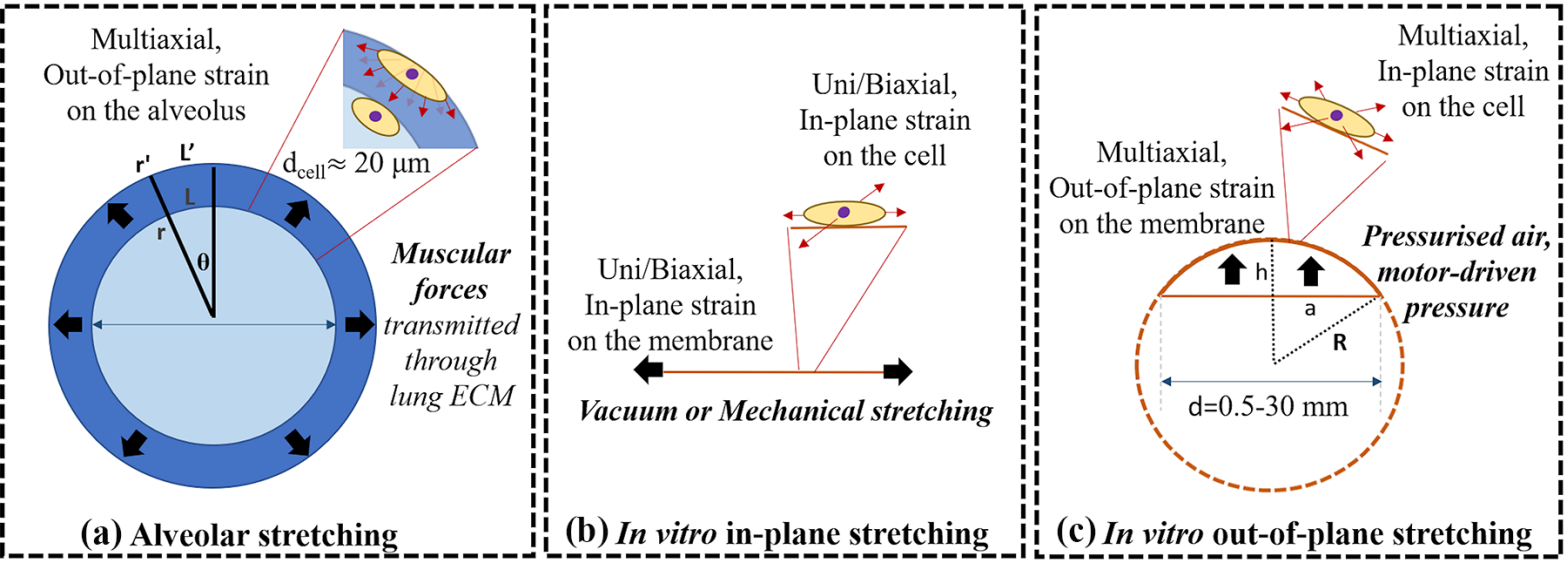
Schematic representation of the: (a) alveolar, (b) in vitro in-plane, and (c) in vitro out-of-plane stretching. Black arrows represent the deformation directions, while red arrows the corresponding strain on the cells.

Ideally (i.e. without considering tissue anisotropy), the alveolus can be considered spherically symmetric, thus each plane passing from its center can be considered a plane of symmetry. Representing the alveolus as an isotropic thin walled sphere (Figure 3(a)), in conditions of small deformation the tangential strain (ε_tangential_) can be defined as:

$$\varepsilon_{tangential}=\frac{L^{\prime }-L}{L}=\frac{r^{\prime }\theta -r\theta }{r\theta }=\frac{r^{\prime }-r}{r}=\varepsilon_{linear};$$

where *L*′ and *L* are the lengths of the arc under the angle θ in stretched and resting conditions respectively. Analogously, *r*′ and *r* are the radii of the alveolus in stretch and resting conditions. Thus, in conditions of isotropic stretching and relaxation, typical of resting state tidal breathing in vivo,^154,156^ the measured linear strain ε_linear_ can be considered the same as that experienced by the epithelial barrier.

Similarly, in vitro systems with 2D circular or semi-spherical shapes as in Figure 3(b) and (c) are symmetric with respect to any plane perpendicular to the membrane rest plane and passing through the center.

The types of deformation applied to the substrate varies greatly but in general can be classified either as out-of-plane or in-plane. The first consists of a deformation characterized by the bulging in/upwards of the substrate (Figure 3(a) and (c)), whereas the second-one is the result of a lateral expansion of the substrate maintaining its original flat position/plane (Figure 3(b)). Furthermore, according to the direction of stress applied and the substrate’s relative displacement due to applied constraints the strains can be uniaxial, biaxial, multiaxial, or radial. This concept is often generalized, or even represented erroneously in the literature, so we provide a basic definition here. In Cartesian coordinates, uniaxial, biaxial, and multiaxial strains respectively occur in the case of a stretching along one, two, or more axial directions, while, in circular configurations, uniform stretching in all the directions along the radius results in a strain which is defined as radial. Tangential or circumferential strains have been also defined in spherical or semispherical out-of-plane deformations, referring to the uniform strains along the surface, perpendicular to the radius. Note that the alveolar barrier undergoes an out-of-plane deformation although linear and tangential strains are equal.

In most devices, the membrane radius ranges from 0.5 up to 30 mm,^157^ while the radius of curvature (*R*) of the membrane can be calculated as follows:

$$R^{2}=a^{2}+{\left(R-h\right)}^{2}\to R=\frac{a^{2}}{2h}+\frac{h}{2};$$

where *a* is the membrane radius and *h* is membrane displacement (Figure 3(c)). For example, for a 12-mm radius membrane, a 5% linear strain is equivalent to vertical displacement of 3.8 mm^158^ and *R* is equal to 20.7 mm. On the other hand, the dimensions of the cell and the radius of curvature of the alveolus are comparable (respectively ≈10 and ≈50 to 100 μm). Therefore, as shown in Figure 3, despite the fact that in vitro models are able to apply linear strains which recapitulate those observed in an alveolus, at the cell scale they generally fail in reproducing out-of-plane deformations. In fact, as demonstrated by recent studies, cells are able to respond to curvatures up to 1000 μm, a phenomenon defined as “curvotaxis,” which may result in cell re-orientation and different gene expression.^159–162^ On typical cell culture systems subject to out-of-plane deformation the effective radius of curvature of the membrane is out of the cell “curvature” sensing range and, consequently, they are likely to “feel” an in-plane deformation. Reproducing the in vivo curvature is important to mimic the cell native environment and the out-of-plane deformations at the cell scale. As the technology hardware for in vitro models improves, it should be possible to investigate this aspect with due attention to assess its importance in modulating cell responses to inhaled substances and therefore include it in the design criteria for physiological relevance.

We should also point out that in vivo and in vitro deformation mechanisms are generally different: in the alveolus, forces generated by the muscles are transmitted through the ECM; while, in vitro, membranes are stretched via pressurized air, vacuum or motor-driven systems, as described further in this section. The stress and strain distributions experienced by the cells may be affected by the deformation mechanism as stress concentrations may appear in correspondence of mechanical constraints, indenters, and membrane fixation points. Additionally, the stress on the alveolar is known to be related to the alveolar pressure level, which varies between −1 and +1 mmHg (101.3 ± 0.1 kPa) during normal breathing. On the contrary, pneumatic pressure devices typically apply pressures up to 7 kPa with respect to atmospheric pressure.^158^ Since it is known that high pressures may damage lung tissues in vivo (e.g. during mechanical ventilation), non-physiological pressure and stress levels are likely to alter cell behavior in vitro.^163,164^

### Pneumatic and motor-driven devices

**Figure 4.**
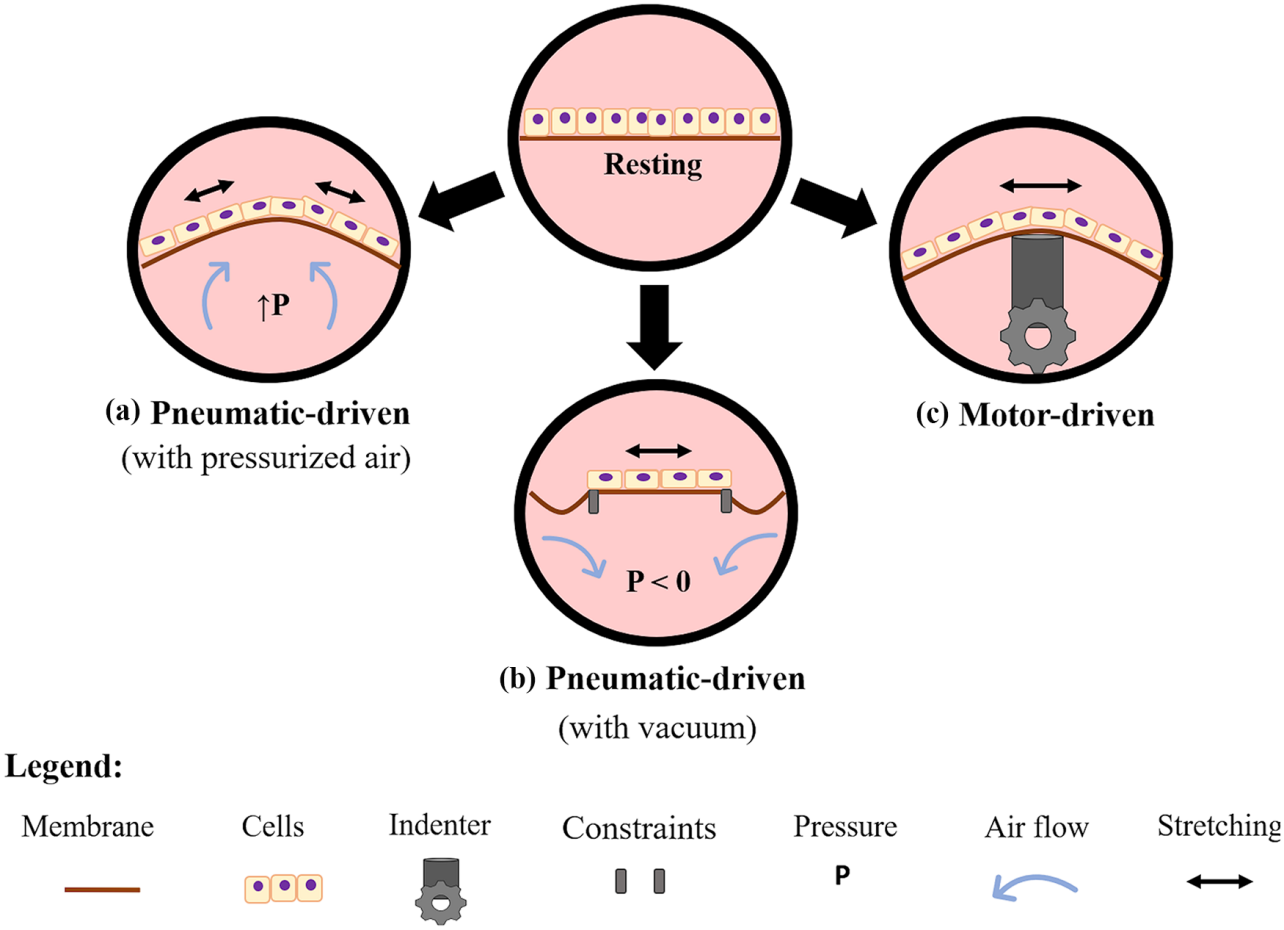
Scheme of the most common principles of actuation for stretching elastic cell culture supports in lung in vitro models. In pneumatic actuation, the support can be deformed either by inflowing air at controlled over pressure (a), or by applying a negative pressure (b), and (c) motor-driven convex surfaces or indenters cyclically deform the support.

Figure 4 schematizes the most common methods for driving stretching motion in in vitro models of the lung. They are based either on the use of pneumatic actuation through the application of air over- or under-pressure or mechanical actuation with indenters.

As shown in Figure 4(a), pneumatic-driven devices deform the culture support using controlled air inflow (i.e. overpressure) or vacuum (i.e. underpressure). The over-pressurization of a chamber upon or underneath a flexible cell culture support is generally achieved thanks to pressure regulators (i.e. electro-valves), which allow the control of air pressure and of the stretching level. These devices appeared from 1989 and were based on precision-cut lung slices^165^ or non-permeable membranes, enabling the application of overpressure under the cells.^166–168^ A more complex device was developed to model bronchiole stretching.^169^ It was able to provide cyclic mechanical strain in combination with ALI. The cylindrical-shaped bronchioles constructed from human lung primary cells were vertically supported by a thin-walled silicone rubber tubing. The device applied mechanical stimulation by pulsing air through the silicone tubing, exerting dilatory forces on the engineered bronchiole. Finally, Cei et al.^158,170^ were the first to combine media flow, ALI, aerosol exposure, and cyclic mechanical strain in a single device to study drug and nanoparticle deposition and passage. Their system, known as MALI (Moving Air Liquid Interface bioreactor), consists of a two-compartment bioreactor with a moving membrane placed between an air-liquid interface, and a nebulizer for quantitative aerosol exposure experiments. In the MALI, an external electro-pneumatic regulator induces an increase of pressure in the apical chamber, while culture medium flows through the basal one; the difference between air pressure and hydrodynamic pressure results in an out-of-plane deformation of the membrane. Notably, MALI and its successor DALI are the first in vitro lung devices to be available as open source technologies.^171^

The second pneumatic-driven approach consists in deforming the cell substrate by applying vacuum underneath a non-porous elastic support (Figure 4(b)), thus they are neither able to model the air-liquid interface, nor to modulate the stretching level. Trepat et al. were among the first to design a cell-stretching system based on this working principle.^172^ Their device consisted in a well with a flexible-bottom a cylindrical loading post located underneath. When a negative pressure was applied under the annular outer region of the substrate, the central area was uniformly stretched, resulting in an in-plane deformation, only in two axes. This type of stretching does not recapitulate the multidirectional out-of-plane deformation that occurs in the alveolar wall. Nevertheless, the stimuli provided allowed the authors to understand how it could modify viscoelastic properties, structural integrity, and micromechanics of human alveolar epithelial cells.^172,173^ Peñuelas et al. used this device to evaluate the antioxidant role of human adult adipose tissue-derived stromal cells when human alveolar epithelial cells were subjected to injurious cyclic overstretching.^174^ Finally, a commercial pneumatic-driven device, is the Flexcell Tension System (Flexcell^®^ International Corporation, Burlington, NC, USA). It allows cell culture on the top of a silicone membrane that is stretched in-plane thanks to a vacuum driven mechanism. Models of lung injury, lung inflammation, or lung tissue repair, as well as changes in cell sensitivity and permeability to compounds and cytokine have been studied with the Flexcell device.^164,175^

As schematized in Figure 4(c), motor-driven systems have been used to deform the cell supports by means of convex surfaces or indenters,^148,176–183^ leaving only one compartment for cell culture. In such systems, stretching level can be tuned by controlling motor displacement. For example, Tschumperlin and Margulies,^148^ Tsuda et al.,^176^ and Cavanaugh and Margulies^177^ used the cyclic movement of a motor-driven annular indenter to deform an elastomeric support (silicone membrane), evaluating the effects of the stretching on alveolar epithelial cells. In detail, the annular indenter contacted the bottom of the silicone membrane near the periphery of the cell culture surface, leading to the sliding of the membrane over the indenter. As result, the membrane stretches transversally with respect to the direction of the indenter motion,^148^ resulting in an in-plane deformation. Tschumperlin and Margulies used this device to study cell vulnerability to different stretching ranges;^148^ Cavanaugh and Margulies showed that applying cyclic stretch with higher amplitudes than the physiological ones led to a decrease of intracellular alveolar epithelial tight junction protein content and to an increase of the permeability.^177^ Finally, Tsuda et al.^176^ showed that the physical stress exerted on the alveolar epithelium by deposited fibrous particulate was greatly enhanced by the tidal cyclic motion of the epithelial cells. A commercial motor-driven device, the Strex cell stretching system (Strex Inc., San Diego, USA), was also developed. Here, cells are cultured within ad hoc designed chambers that are clamped both to a fixed frame and to a movable frame, which moves by connection to a stepping motor, leading to a uniaxial in-plane deformation of the seeding support. This system was used by Ito et al.^184,185^ to investigate the effects of mechanical stretch in pulmonary endothelial cells or airway smooth muscle cells.

Finally, Choe et al.^186^ designed a bioreactor system able to apply a mechanical cyclic stretch combined with ALI to characterize the effects of dynamic compression in ECM remodeling in a physiologically relevant 3D environment. The stretching device presented individual wells with movable inner walls designed to introduce lateral compressive strain, leading to an in-plane deformation of the substrate where cells were seeded. Cyclic compressive strain was imposed via a motor-driven mechanical arm. This device was also used by Tomei et al. to evaluate the effects of dynamic compression on lentiviral transduction in an in vitro airway wall model.^187^

Some alternatives to pneumatic- and motor-driven approaches have been also used for lung cell stretching.^144,188–194^ For instance, Skinner et al.^144,192–194^ used a solenoid unit to stretch a substrate seeded with cells. The substrate was fixed to a dish at one end and to a moving iron bar at the other end; the alternating electromagnetic field generated caused the iron bar to move back and forth, deforming the support on which cells were cultured.

In summary, the integration of membrane actuation systems in bioreactors has enabled cyclic movements reminiscent of breathing in vitro. Although the majority of these systems are capable of mimicking physiologically relevant (linear and tangential) strain levels, they fail in reproducing the actuation mechanism and the cyclic change in curvature at cell scales.

## Lung-on-chips: Breathing at the microscale

The dimensions of engineered systems described in the previous section were comparable to those of traditional culture plates (multiwells, transwells, etc.), facilitating the transfer of cell culture protocols. But a big step in downscaling has taken place in recent years, since the design of complex microscaled fluidic devices, known as organ-on-chips, took off.^195^ Polydimethylsiloxane (PDMS),^196^ a well-known transparent, biocompatible, and easily moldable silicone, is commonly used to fabricate these devices by soft-lithography. These small chips possess microfabricated microchannels that can be continuously perfused and lined with living cells.^197,198^ Lung-on-chips have been thus proposed for drug testing, toxicology studies, and disease modeling.

Nalayanda et al.^199^ were the first to report a lung-on-chip platform in 2009 in the form of a miniaturized ALI set up. Media flow on the basal side guaranteed the nourishment of cells, while an open system on the apical side exposed the cells to air. Using this chip, they assessed the integrity and functionality of A549 monolayers. Some years later, Long et al.^200^ presented a similar device: the authors designed a two-chamber system to accommodate commercially available cell culture membrane supports. In this case, computational simulations were run to optimize the chip design and maximize gas transport on the liquid side of the alveolus.

Aiming at integrating more than one cell type in a chip, in 2015 Benam et al. described a microscaled system that hosted the co-culture of differentiated, mucociliary bronchiolar epithelium on the air side, and an underlying microvascular endothelium exposed to fluid flow.^201^ It was used to model complex and dynamic inflammatory responses of healthy and diseased lungs in vitro. A similar model was proposed by Jain et al.,^202^ with the difference that cells of the endothelial side experienced whole blood flow instead of cell culture media flow, allowing a quantitative analysis of inflammation-induced thrombosis. Still co-culturing epithelial with endothelial cells, this time together with pulmonary fibroblasts, Barkal et al.^203^ developed a microscale organotypic model of the human bronchiole for studying aspergillosis.

Showing that chips can also be used for exposure studies in the field of inhalation toxicology, Benam and co-workers reported a system that integrated a lung-on-chip microfluidic with a smoke generator and a micro-respirator that recapitulates human smoking behavior.^204^ This chip permitted the analysis of the effects of whole smoke, from both conventional tobacco and electronic cigarettes, delivered under physiologically relevant flow conditions.

With a completely different application, Li et al. used a lung-on-chip for studying long-term electrotaxis,^205^ evaluating cancer cell re-orientation and migration directionally under a physiological electric field. Their device did not present an ALI, but a simple cell culture channel divided into three segments of different widths, in order to allow the investigation of electrotactic migration.

### Stretching lung-on-chips

**Figure 5.**
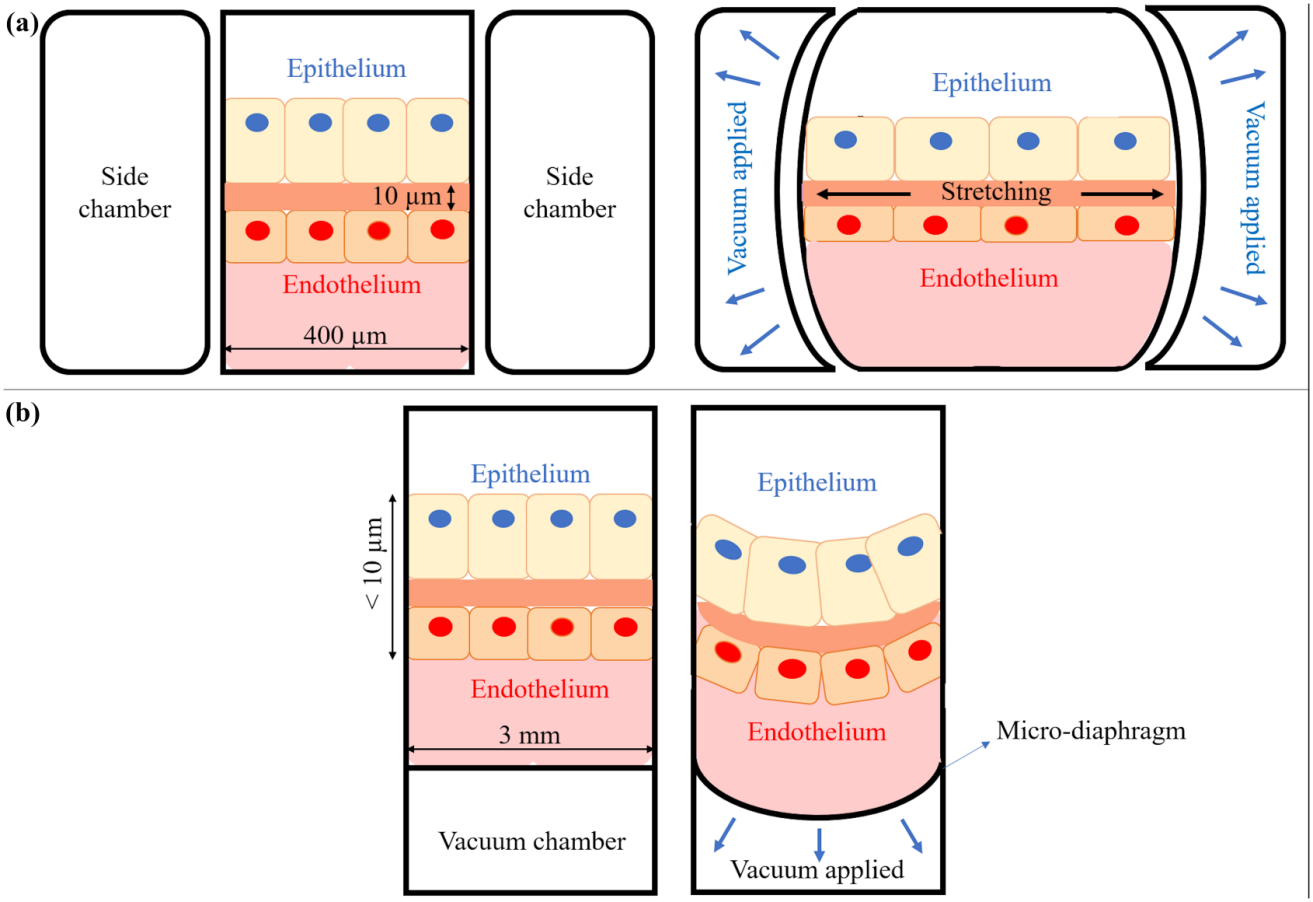
Schematic representation of two different working principles of breathing chips: (a) Huh et al.^207^ working principle: the microfabricated device uses compartmentalized PDMS microchannels to mimic the lung breathing sequence, and (b) Stucki et al.^213,214^ working principle: a micro-diaphragm actuated by an electro-pneumatic set-up leads to the cyclic motion of the cells.

Despite being fairly complete and versatile devices, none of the chips mentioned above reported a mechanical stimulation of the cells able to reproduce breathing movements. Such functionality first appeared in 2008, in a device described by Kamotani et al.,^206^ and took a turning point in 2010 with the widely publicized chip developed by Huh et al.^207^ Kamotani et al’s. device was an array of miniature cell stretching chambers that enabled the study of the effects of mechanical strain in a parallel manner amenable to higher throughput screening.^208^ The system used microwells with flexible bottom membranes that were placed over piezoelectrically actuated pins that pushed against a membrane seeded with cells, applying an out-of-plane deformation of the seeded support. Huh et al. developed a lung-on-chip device able to apply a cyclic mechanical stretch to the cells.^196,207–210^ This microfabricated two-channel system employs the vacuum actuation method used in the FlexCell (see section on pneumatic devices above), but with a different configuration: the membrane is attached to a flexible frame placed between two chambers, the upper one for the airflow, and the bottom one for the media flow. The vacuum channels are located on the side and when vacuum is applied the frame moves leading to an in-plane uniaxial deformation of the membrane (Figure 5(a)). The device has been used for a number of applications: as an alveolar-capillary mimic to simulate bacteria and inflammatory cytokine responses^207^; in the nanotoxicology field to evaluate how cyclic mechanical stretching affects toxic and inflammatory response to silica nanoparticles^207^; in disease modeling and therapeutic substances studies, predicting the activity of a drug for pulmonary edema^210^ and recapitulating lung cancer growth, tumor dormancy, and responses to tyrosine kinase inhibitor therapy.^211^

Some microchips have been designed to couple different compartments that represent different organs, to study how they communicate. For instance, Liu et al. studied brain metastasis,^212^ by connecting an upstream “lung” with a downstream “brain,” characterized by a functional blood–brain barrier structure. The lung part of this microdevice was actuated like Huh’s chip.^209^ The concept of stretching lung-on-chips has been taken up by several teams, exploring other ways of actuating flexible substrates. An interesting device known as the breathing lung-on-chip device with a new design was fabricated by Stucki et al. in 2015.^213,214^ This chip was able to reproduce the cyclic out-of-plane motions that occur during breathing thanks to a micro-diaphragm that was actuated by an electro-pneumatic set-up. The fluidic part of the chip consisted of cell culture wells with porous and flexible membranes, while the micro-diaphragms were integrated into the pneumatic part and connected to pneumatic microchannels. Applying vacuum underneath the diaphragm led to its displacement and membrane motion, as shown in Figure 5(b). Given the dimensions of the device, it is the only system which reproduces a relevant out-of-plane deformation at cell scales. This chip was used to evaluate permeability properties of epithelial cell layers and to demonstrate that cell strain influences the metabolic activity and the cytokine secretion of primary human pulmonary alveolar epithelial cells obtained from patients. Felder et al. used a chip with the same diaphragm-like actuation to examine the influence of mechanical strain on alveolar epithelial wound healing in idiopathic pulmonary fibrosis.^215^ Another chip design for an out-of-plane deformation of the cell substrate by means of pneumatic actuations was that of Campillo et al.^216^ One of the main differences of this system, compared with Stucki’s chip, is that the flexible membrane deflects upwards by cyclically increasing gas pressure beneath it. Interestingly, Campillo’s device was used for a novel application: to study the effects of intermittent hypoxia, a hallmark of obstructive sleep apnea.

To sum up, the chips designed by Kamotani and Campillo are able to apply a cyclic deformation to the substrate, even though they do not present ALI and media flow. These features are instead present in both Huh’s and Stucki’s chips.^207,213^ Nevertheless, none of the mentioned devices presents a system to directly expose cells to compounds or NPs for allowing quantitative aerosol exposure experiments.

## Discussion

In this paper we overview existing lung in vitro models starting from the simplest static models up to more elaborate engineered systems that better reproduce the mechanophysical stimuli present in vivo.

Although they have proven useful, the traditional static models whether cultured at ALI, and/or in co-culture, or even arranged in 3D, are not fully representative of the complexity of the dynamic lung environment. One side of this aspect has been addressed through the use of fluidic systems, such as bioreactors or microfluidic chips in combination with ALI culture (Table 5). Interestingly, most of the studies reported in Table 5 concern two main applications: inhalation toxicology and aerosol drug delivery testing. In this context, the ability to aerosolize particles is a must for investigating the interactions between cells and inhaled particles, as is the ability to accurately dose the amount of material coming in contact with the cells.^217^ As discussed in the section on exposure systems, there are commercial devices that meet such characteristics and are becoming standards for toxicology, including the testing of cigarette and tobacco products, since in these cases a high level of reproducibility is demanded. Moreover, the requirement for reproducibility reflects the favored use of commercial 3D cell models in several of the studies reported in the section on exposure systems and summarized in Table 4. Nevertheless, although there is a choice of commercial platforms to perform exposure studies, the effects of external aerosolized compounds are also mediated by factors that these systems cannot reproduce, such as the rhythmic contraction during the breathing. Hence the development of engineered systems that apply mechanical stimuli to cells is still a growing research field. Table 6 summarizes the systems that provide mechanical stretching. Pneumatic and motor-driven actuation are the main methods to stretch cell culture substrates, although microfluidic systems are also becoming widespread. Indeed, the first microchips posed a lurking “competition” to the milli-scaled devices present at the time, since these micro-platforms are a versatile solution for different applications, as shown in Tables 5 and 6. However, despite the advantage of requiring a low amount of material and space, which may allow the integration of multicompartmental models in a single chip, microscaled devices present several drawbacks. As reported by Mattei et al.^218^ these systems provide high wall shear stress, due to the high surface to volume ratio, and are subjected to *edge-effects*, since a large portion of cells lie at the periphery of the system and do not interact properly with other cells. They are also known to be tricky to handle, requiring a great deal of patience and expertise.^219^

**Table 5.** Systems that apply a media flow (dynamic systems) or combine ALI with aerosol/smoke exposure.

Scale	Type of product		Medium flow	ALI	Aerosolization	Applications	Examples
**Macro**	Commercial	No exposure	Yes	Yes	No	Organoids for personalized disease modeling, tissue engineering, evaluation of biofilm formation	Synthecon bioreactor
						Toxicology	Cultex LTC-C
		ALI+exposure	Yes	Yes	Yes	Toxicology, evaluation of drugs and cancer mechanism	Cultex RFS
							Vitrocell exposure chamber
							PreciseInhale
						Smoke exposure—toxicology	Vitrocell Smoking machine
							Borgwaldt Smoking machine
	Laboratory-made systems		No	Yes	Yes	Toxicology	Bakand et al.,^45^ Blank *et al*.,^51^ Riediker et al.,^46^ Switalla et al.,^44^ Cooney et al.,^47^ Holder et al.,^48,49^ Oosting et al.,^50^ Rothen-Rutishauser et al.,^54^
						Study the deposition and transport of inhaled drugs	Fiegel et al.,^57^ Cooney et al.,^58^ Haghi et al.,^56^ Grainger et al.,^59^
						Toxicology, electrostatic precipitation mechanisms	Savi et al.,^52^ Stevens et al.,^53^ Volckens et al.,^67^ De Bruijne et al.,^63^ Stoehr et al.,^65^ Holder et al.,^64^ Frijns et al.,^42^ Hawley et al.,^66^
			Yes	No	No	Cell-NP interaction	Breitner et al.^34^
				Yes	No	Patho-physiological stretching models, stem cell differentiation	Grek et al.,^26^ Jeans et al.,^32^ Ghaedi et al.,^27^ Blume et al.,^33^ Box et al.^31^
					Yes	Toxicology	Tarkington et al.,^43^ Tippe et al.,^60^ Bitterle et al.,^61^, Lenz et al.^62^
**Micro**	Lungs-on-chips		Yes	Yes	No	Evaluation of chip efficiency, design optimization of liquid-phase flow patterns, long-term electrotaxis study, disease models	Nalayanda et al.,^199^ Long et al.,^200^ Benam et al.,^201,204^ Li et al.,^205^ Jain et al.^202^
						Lung inflammation mechanisms	Barkal et al.^203^
					Yes	Analysis of the effects of whole smoke	Benam et al.^204^

**Table 6.** Systems that apply mechanical stretching.

Scale	Actuation method	Flow	ALI	Co-culture	3D	Authors/commercial system	Strain type	Strain range
**Macro**	Pneumatic	No	No	No	No	Winston et al.^167^, Gorfien et al.^166^, Pugin et al.^168^	Multiaxial, out-of-plane	0%–15%
						Trepat et al.^172,173^	Biaxial, in-plane	0%–20%
					Yes	Dassow et al.^165^	Multiaxial, out-of-plane	10%–25%
				Yes	No	Peñuelas et al.^174^	Biaxial, in-plane	15%
			Yes	No	Yes	Miller et al.^169^	Multiaxial, out-of-plane	2%
		Yes	Yes	No	No	Cei et al.^158^	Multiaxial, out-of-plane	5%–17%
				NA	NA	FlexCell (commercial)^163^	Biaxial, in-plane	8%–22%
	Motor-driven	No	No	No	No	Tschumperlin et al.^148^, Tsuda et al.^176^, Cavanaugh et al.^177^	Multiaxial, in-plane	0%–25%
						StrexCell (commercial)	Uniaxial, in-plane	0%–30%
			Yes	Yes	Yes	Choe et al.^186^, Tomei et al.^187^	Uniaxial, in-plane	0%–30%
**Micro (lungs-on-chips)**	Pneumatic	No	No	No	No	Campillo et al.^216^	Multiaxial, out-of-plane	0%–20%
		Yes	Yes	No	No	Felder et al.^215^	Multiaxial, out-of-plane	0%–20%
				Yes	No	Stucki et al.^213,214^	Multiaxial, out-of-plane	10%
						Huh et al.^207,209,210^, Hassel et al.^211^, Liu et al.^212^	Uniaxial, in-plane	10%
	Motor-driven	No	No	No	No	Kamotani et al.^206^	Multiaxial, out-of-plane	0%–25%

NA: not applicable.

Clearly, the literature from the past 20 years in engineering lung in vitro models describes remarkable progresses, but as yet these advanced systems fail in fully recapitulating the in vivo environment. To date, the main issues are related to mimicking: (i) the alveolar architecture (dimension, spherical structure, and interconnection with adjacent alveoli in the acinus and with the capillaries that surround the alveolus), which may affect aerosol deposition, transport, and cell stretching; (ii) mechanical properties (e.g. membrane elastic and viscoelastic properties), which may influence cell behavior; (iii) biochemical properties related to the presence of a surfactant layer that, besides avoiding alveolar collapse or hyperextension, is likely to interfere with the passage of substances (and pathogens) across the cell barrier.^220^

It is well known that micro-scale extracellular matrix properties strongly influence cellular growth, migration, and differentiation, as well as cellular response to mechanical and biochemical signals.^221–226^ This aspect is true for every organ type, but there are tissues whose function hinges on their intricate structures, and this is the case of the alveoli. Addressing the issue of alveolar architecture, several authors are focusing their efforts in building materials for generating complex 3D structures able to recreate these biophysically and biochemically entangled networks. In this direction, Grigoryan et al.^227^ used stereolithography to build soft hydrogels containing such biomimetic and multivascular architectures. They managed to print a bioinspired alveolar model with an ensheathing vasculature, which was also able to sustain a cyclic ventilation with humified oxygen gas, maintain the viability of mammalian cell lines, and support the normal function and differentiation of primary human stem cells. This work represents an important step forward in combining an alveolar-like architecture with the cyclic stretching movement that mimics breathing. However, work still needs to be performed in order to have a coherent approximation of scalable lung-specific design. With the goal of obtaining in vivo-like structures, also Erben et al.^228^ used stereolithography to print mm-sized high precision 3D scaffolds at micrometer resolution.

As far as mechanical properties are concerned, as demonstrated in the section describing alveolar motion, despite the fact that stretching devices are able to apply (patho)physiological strain levels, they are not able to fully replicate the three-dimensional nature and scales of alveolar stretching. Indeed, in most of the cases the systems provide an in-plane stretch and, also in the case of out-of-plane stretching, membrane fixation, constraints, and indenter contact points likely result in non-uniform (and hence difficult to characterize and control) strain distributions. However, it should be noted that the assumption of isotropic breathing may be an oversimplification of the in vivo dynamics, which is probably affected by intrinsic tissue anisotropy. Another crucial mechanical aspect which is often overlooked is the elastic modulus of the cell culture substrate compared with the lung, which is a highly stretchable soft tissue with an elastic modulus of the order of 3 kPa. Most of the materials used as flexing substrates, such as PDMS, are very stiff with elastic moduli of the order of megapascals. Thus, the forces or pressures required to deform the substrates are much higher than experienced in the alveoli.

As a result, devices with mechanical stimuli comply at different levels with the engineering requirements mentioned in the Introduction. They therefore dictate distinct applications (or restrictions on the application, if we may say). In fact, most of the stretching devices presented in Table 6 do not provide media flow, and even in the cases where technical solutions were adopted to culture cells at ALI, aerosol exposure was not contemplated. The concomitant presence of cell stretching, flow, and a reproducible aerosol exposure system definitely poses an engineering challenge. Up to date such achievement has been reached in only one device at the “milliscale.”^158^ The present challenge of engineered lung models is the design of the “all-in-one device,” which combines all the features existing in the lung (i.e. lung architecture, stretching movement, and aerosol exposure). This is the direction in which many research efforts are pointed, with the prospect of replacing, at least partially, animal models with in vitro models. However, not all studies need a holistic approach. When engineering an in vitro model, its application should set the requirements of the design. For example, when modeling lung tissues (e.g. bronchi) that differ from the alveolus—which is the functional unit that deforms during the breathing—it is not necessary to mimic cyclic mechanical stretch; while in applications that do not foresee toxicological studies or testing the impact of inhaled substances, an exposure chamber will not enhance the reliability of the model. Therefore, when developing a lung model, the key is to identify which relevant physiological parameters should be reproduced according to the research question being addressed and the context of the future experiments. Similarly, lung device-users need to define the application and then choose the device accordingly.

Another important point which needs to be addressed is the possibility to monitor or interrogate the cells during culture, performing the measurement necessary for the study. While media collection for different cell assays is usually enabled by the presence of valves in the fluidic circuits or in the culture chambers, other measurements can be a challenge. For example, evaluating barrier integrity is fundamental for passage studies. However, although the presence of an intact barrier can be visually monitored in a qualitative manner, not all the devices are optically transparent and compatible with microscopes. Quantitative information can be obtained using transepithelial electrical resistance/impedance (TEER/TEEI) measurements, but they are not easily integrated at ALI. Therefore, further efforts are also needed to develop efficient and non-invasive monitoring systems for the evermore sophisticated devices we engineer.^28^

After overcoming the technical challenges of developing advanced cell culture tissues, there are still other hurdles to face. Naturally, the acceptance of these devices for the day-to-day use in common laboratories might not be easy. To overcome a possible resistance two approaches are crucial: engineers should work in close collaboration with the final users (biologists, toxicologists, among others) of the device in its development phase; make the device compatible as much as possible with common lab instruments and assays. In this way, the validation of the system becomes a similar process to that of testing any other new practice/assay. Positive and negative controls, as well as multiple replicates, are essential for the verification of the results. In the context of testing a bioreactor, for instance, it would consist of first testing individually each of the dynamic cues (taken then as variables) the system can provide. On the same line of thought, it is advisable to start using the devices in simple context and for small experiments, rather than adding too many variables to the set-up. From a biological point of view, researchers might want to consider starting by culturing more robust and reliable cell sources, such as cell lines, to perform the proof of concept of the device. Once this is accomplished, other cell types and more complex cellular arrangements can be included in the protocol. Ideally, in a further step, different labs and research groups would have the opportunity to test their protocols on the new system.

On a final note, the future direction of lung in vitro models will depend greatly not only on the upcoming technologies in the engineering field, but also on the ever-changing motivations to use them. This aspect appears even more clear nowadays when the COVID-19 pandemic highlights the importance of having ad hoc reliable and predictive in vitro models for a systematic study of respiratory diseases. Interestingly, the pandemic has also brought home the impact of open-source technologies for rapid and efficacious solutions to biomedical emergencies.^229^ Although many of the in vitro devices described in this review are commercial systems, there is still plenty of scope for new developments based on open-source collaborative design which may help address some of the issues such as mimicking lung complexity in a simple to use system, handling, and non-destructive intermediate and end point analysis.
